# Do variations in the theatre team have an impact on the incidence of complications?

**DOI:** 10.1186/1471-2415-6-13

**Published:** 2006-03-16

**Authors:** Oliver J Baylis, Wendy E Adams, David Allen, Scott G Fraser

**Affiliations:** 1 The Department of Ophthalmology, Sunderland Eye Infirmary, Queen Alexandra, Road, Sunderland, SR2 9HP, UK

## Abstract

**Background:**

To examine whether variations in non-medical personnel influence the incidence of complications in a cataract theatre.

**Methods:**

A retrospective Case-Control study was undertaken in a single-site, designated cataract theatre. Staffing variations within theatre were examined and the incidence of cataract complications was assessed.

**Results:**

100 complicated lists and 200 uncomplicated control lists were chosen. At least 7 nurses were present for every list. Mean experience of the nurses was 6.4 years for case lists and 6.5 years for control lists. Average scrub nurse experience in years was 7.6 years for complicated lists and 8.0 years for controls. 26% of complicated case lists were affected by unplanned leave and 17% in control lists. Odds ratio 1.7 (1.0 to 3.1) 95% CI.

**Conclusion:**

Unplanned leave can have a detrimental effect on the operating list. The impact of this may be modifiable with careful planning.

## Background

Cataract surgery is the commonest elective surgical procedure performed in the UK [1]. As techniques and equipment have advanced, the chances of a good outcome have improved and the complication rate has fallen [2]. However, when complications do occur they can have serious consequences.

One of the commonest sight threatening intraoperative complications is tearing of the posterior capsule of the lens [1]. When this happens there may be a need for further surgery, a higher risk of severe infection and a worse visual prognosis [3-5]. The mean incidence of capsule tear in phacoemulsification is 4.4% (range 0.7–16) [6,7].

Although a wide range of complications can occur during cataract surgery, we have found in previous work that posterior capsule rupture can be a useful indicator of problems during the course of the operation [8].

**Figure 1:**
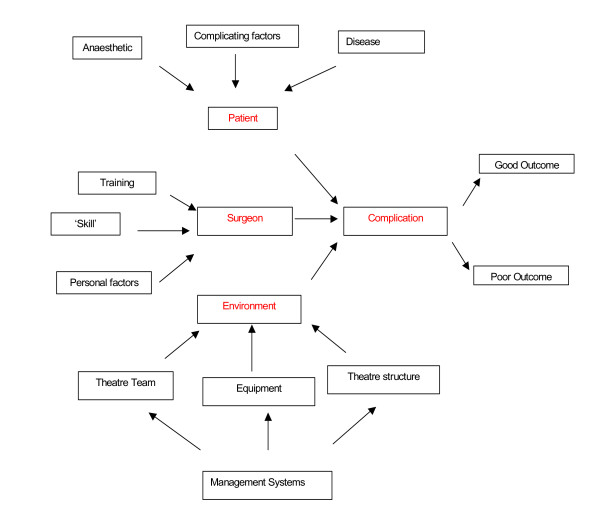
the reasons for surgical complications are likely to be multifactorial.

When a patient sustains a complication, in any field of medicine, the cause is likely to be multifactorial and some of these interlinking causes are represented in Figure 1.

Previous work has tended to concentrate on operating techniques, equipment or surgical skill and there has been far less work looking at indirect influences on the surgeon – such as the variations in the non-medical members of the theatre team [9]. This study was designed as an attempt to examine the influence of some of these variations.

## Methods

A retrospective case-control study was undertaken in a designated theatre (i.e. only cataract extractions) at a single site, dedicated eye hospital between January 2001 and December 2003. Seven consultant eye surgeons operate in this theatre each with his/her own theatre team (although no nurse works exclusively for one surgeon). The standard nursing team consists of a scrub nurse, a nurse accompanying each patient through the operation and one floor nurse. One nurse from each category is involved in each operation but the total number of nursing staff involved per list varies depending on the total number of cases on the list e.g. in a higher volume list there may be 2 alternating scrub nurses and up to 6 nurses rotating to accompany the patients. In this theatre, all non-medical staff are nurses i.e. there are no porters or operating department assistants and all are registered nurses.

One hundred consecutive operating lists containing at least one complicated cataract operation were identified from the theatre logbook and confirmed by the hospital electronic database. Inclusion criteria, for the purpose of our study, were cataract operations complicated by posterior capsule tear with or without anterior vitrectomy. Exclusion criteria were operations not done by consultant surgeons and those with other complications. Two uncomplicated operating lists, as near as possible to the date of the case list, were chosen as controls and matched for surgeon, day of the week, session time, and technical difficulty (as defined by a preoperative score[8]).

The nurses involved in each operation on the case and control lists were identified from the hospital electronic database and these details were crosschecked with the nursing duty rota. For each operating list the following factors were assessed:-

1. The average number of nurses present in theatre for the list

2. The cumulative nursing experience in years recorded for each operating list (recorded from the start date in the cataract theatre at our hospital).

3. The average nursing experience in years per operating list

4. The experience in years of the scrub nurse recorded for each case and control list.

5. The numbers of nursing staff absent from work with unplanned leave i.e. short notice absences (usually on the actual day) either sick or compassionate leave.

## Results

100 lists with complications and 200 uncomplicated control lists were identified. The results are shown in Table 1. The average number of nurses present per list was 7.4 for the complicated lists and 7.3 for the control lists. The cumulative experience of the nurses per list was 47.4 years for complicated lists and 48 years for control lists. The average experience of a nurse per list (cumulative experience / number in theatre) was 6.4 years for the complicated lists and 6.5 years for control lists. Average scrub nurse experience was 7.6 years for complicated case lists and 8.0 years for equivalent control lists.

**Table 1:** Profile of nurses present in theatre for lists with complications (Cases) versus those without (controls).

Variable	Cases (100)	Controls (200)	
Average number of nurses per operating list	7.4	7.3	
Cumulative experience per operating list (yrs)	47.4	48.0	
Average nurse experience per operating list (yrs)	6.4	6.5	
Scrub nurse experience (yrs)	7.6	8.0	
Number of lists affected by unplanned leave	26	34	Odds ratio = 1.7 (1.0 to3.1) 95%CI

According to the duty rotas, there were nurses absent with unplanned leave in 26 of the lists with complications (26%) and 34 absent in the control lists (17%). There were therefore more complications in the lists with unplanned leave amongst staff. We calculated the odds ratio of the risk of complications in lists with unplanned leave as 1.7 (1.0 to 3.1, 95% confidence interval) (Table 1)

The **total number **of staff present in theatre was not affected by unplanned leave as staff were drafted into theatre from other duties.

## Discussion

The aim of this study was to investigate whether fluctuations in numbers and experience of the non-medical theatre team were associated with variations in the incidence of intraocular complications. The following factors were considered:-

### 1. Number of nurses present per list

It was found that the number of nurses in theatre per operating list was similar in case and control groups (7.4 and 7.3). This study did not show that the number of nurses present contributed to the incidence of complications.

### 2. Experience of nurses

The experience of nurses was similar in both the complicated and control lists. It is often thought that more experienced staff are associated with a lower incidence of complications, which may be true, but in this designated cataract theatre there is an overall high level of experience (78% of nurses have 4 or more years experience in this cataract theatre). In addition there has been a relatively low level of staff turnover which maintains this high level of expertise in cataract surgery. This study did not show that there was a significant difference in levels of experience between complicated and control lists.

### 3. Experience of scrub nurse

The average experience of the scrub nurse involved in a complicated case was almost identical to that of a scrub nurse for a control case (7.6 and 8.0). This again probably reflects the high level of nursing experience and low staff turnover rate in this particular theatre.

### 4. Unplanned absence

In total, 19% of the operating lists were affected by unplanned leave. Unplanned leave occurred more often in complicated (26%) than uncomplicated (17%) lists (odds ratio 1.7, 95%CI 1.0 to 3.1). Our results seem to suggest a significant influence of unplanned leave on complications.

As indicated from the other results, this was not because of a reduction in total number or experience of the replacement theatre staff suggesting that there must be other influences. When a theatre nurse is unexpectedly absent, the nurses who were originally assigned to other tasks for the session such as clinics, pre-op assessments, post-op checks are drafted into the theatre at short notice. Perhaps this in itself may disrupt the normal equilibrium of the operating theatre. If this is what occurred it could be for a number of reasons:-

1. Individual effect – the physical/psychological act of being moved unexpectedly from one task to another is stressful and rapidly changing role may be difficult.

2. Team effect – changing the designated theatre team for that list may lead to:

• Interpersonal stresses e.g. surgeon and a new scrub nurse that normally does not work in this team

• Unusual combinations of staff with unrecognised difficult interactions

• Other subtle resonances that are more difficult to identify

These factors added together could lead to overt or hidden discord in the operating theatre creating tensions that do not cause complications in themselves, but may create situations where they are more likely to occur.

The results from our study have potential relevance in other surgical settings and indeed have already been identified to some extent. In the field of laparoscopic surgery, it has been shown that the use of a designated, specialist theatre team led to a reduced operating time and a lower conversion rate to open surgery[10]. In addition, if a laparoscopic surgical team establishes a specific routine for the operating list this leads to a less stressful working environment [11].

A recent review by the Commission for Healthcare Audit and Inspection examined the current situation in ward staffing, and found a high incidence of nursing absence, especially due to sick leave [12]. The clinical consequences of nursing fluctuations in this situation are measurable, for example, by incidence of pressure sores, hospital acquired infections and adverse clinical incidents. In theatre, however, the clinical outcome is often measured by the results of the operation alone, which makes it difficult to gauge whether or not staffing levels in theatre have had an affect. Staff sickness absence rates in day surgery generally are published as 5.6% up to 25% [13]. The review of day surgery, published by the Commission for Healthcare Audit and Inspection attributed some cancellations of theatre lists to unforeseen staff absence, but staff absence was not examined with relation to outcome of surgery [14].

### Potential biases

The design of the study was constructed to match case and control operating lists as far as possible, in terms of factors such as operating surgeon, cases per list, day of the week, time of lists and case mix – factors which we took from the results of our previous work [15]. However, other factors may also influence whether or not a complication occurs, and matching lists in this way can never eliminate all of these. Quality of data collection relies on the entries on the computer system and on the nursing rotas. These were crosschecked as far as possible, but this is also a potential source of bias. It is difficult to accurately describe the theatre conditions for any given operation looking solely at these parameters and very difficult to measure the theatre team's effectiveness. Posterior capsule tear was chosen as the measurable complication because it is:-

1. Easily recordable

2. Potentially sight threatening, but not rare (posterior capsule rupture published incidence 4% compared with endophthalmitis < 0.14%) [16].

3. Representative of intraoperative problems i.e. it cannot happen pre- or post operatively.

We feel that it is a useful complication to allow retrospective examination of the operating environment but we recognise this is only one type of complication and other complications may also have to be considered.

The results from this study are relevant to all operating theatres especially when planning staff levels and mix of experience. Our study has suggested that complications do not happen simply because of a reduction in staffing numbers but when the normal theatre team are disrupted by unplanned leave.

As far as we are aware this is the first time that this effect has been documented. If our results are confirmed within other specialities and hospitals we would propose that those responsible for theatre nursing rotas factor in our findings. They could do this by appointing a designated replacement for each theatre nurse from those working outside theatre on that day. This would mean that the replacement nurse would:-

1. Be aware they may have to go into theatre if their 'partner' were unexpectedly absent.

2. Allow the rota administrator to, as far as was practical, match the replacement nurse for experience and to use their knowledge of working relationships with other nurses in that theatre and the operating surgeon for that list.

Although we are aware that this may not be practical in some units, it may well be a strategy that would enable lists to proceed with less disruption and would reduce confusion and anxiety in the event of unexpected leave. Our study suggests that this may reduce the likelihood of complications in these disrupted lists.

## Conclusion

There was no evidence of a difference between the numbers in theatre, or in the experience of non-medical staff when comparing complicated and uncomplicated operating lists. Operating lists affected by unplanned leave, however, showed a higher incidence of complications when compared to control lists. It would therefore seem prudent to have a strategy for dealing with the event of unplanned leave thereby reducing the likelihood for error.

There are limitations to this study, however, and similar studies (ideally prospective) need to be undertaken to verify results in different centres and within different specialties.

## Competing interests

The author(s) declare that they have no competing interests.

## Authors' contributions

OB and WA gathered the data. SF designed the study. OB, WA, EDA and SF analysed the data and wrote the paper.

## Pre-publication history

The pre-publication history for this paper can be accessed here:



## References

[B1] Cataract Surgery Guidelines (2004). Scientific Department, The Royal College of Ophthalmologists.

[B2] Arbisser LB (2004). Managing intraoperative complications in cataract surgery. Curr Opin Ophthalmol.

[B3] Wong TY, Chee SP (2004). The epidemiology of acute endophthalmitis after cataract surgery in an Asian population. Ophthalmology.

[B4] Miller JJ, Scott IU, Flynn HW, Smiddy WE, Newton J, Miller D (2005). Acute- onset endophthalmitis after cataract surgery (2000–2004): incidence, clinical settings, and visual outcomes after treatment. Am J Ophthalmol.

[B5] Onal S, Gozum N, Gucukoglu A (2004). Visual results and complications of posterior chamber intraocular lens implantation after capsular tear during phacoemulsification. Ophthalmic Surg Lasers Imaging.

[B6] Desai P, Minassian DC, Reidy A (1999). National cataract surgery survey 1997–8: a report of the results of the clinical outcomes. Br J Ophthalmol.

[B7] Vajpayee RB, Sharma N, Dada T, Gupta V, Kumar A, Dada VK (2001). Management of posterior capsule tears. Surv Ophthalmol.

[B8] Habib M, Mandal K, Bunce CV, Fraser SG (2004). The relation of volume with outcome in phacoemulsification surgery. Br J Ophthalmol.

[B9] Oetting TA (2003). Preventing and managing cataract complications: it takes a village. Insight (American Society of Ophthalmic Registered Nurses).

[B10] Kenyon, Lenker MP, Bax TW, Swanstrom LL (1997). Cost and Benefit of the Trained Laparoscopic Team. Surg Endosc.

[B11] Winer WK, Lyons TL (1995). Suggested set-up and layout of instruments and equipment for advanced operative laparoscopy. Journal of the American Association of Gynecologic Laparoscopists.

[B12] Acute Hospital Portfolio Review (2005). Ward Staffing. Commission for Healthcare Audit and Inspection.

[B13] Tanner J, Bailey G (2001). Staff Rostering in the Operating Department. Br J Perioper Nurs.

[B14] Acute Hospital Portfolio Review (2005). Commission for Healthcare Audit and Inspection.

[B15] Kamalarajah S, Silvestri G, Sharma N, Khan A, Foot B, Ling R, Cran G, Best R (2004). Surveillance of endophthalmitis following cataract surgery in the UK. Eye.

